# Natural Ventilation and Air Purification for Effective Removal of Airborne Virus in Classrooms with Heater Operation

**DOI:** 10.3390/toxics10100573

**Published:** 2022-09-30

**Authors:** Su-Hoon Park, Se-Jin Yook, Hyun Bon Koo

**Affiliations:** 1 School of Mechanical Engineering, Hanyang University, Seoul 04763, Korea; 2 Department of Structural Engineering Research, Korea Institute of Civil Engineering and Building Technology, Goyang-si 10223, Korea

**Keywords:** natural ventilation, air purifier, airborne infection, atmospheric aerosol, virus transmission, classroom ventilation, indoor air quality, indoor environment, aerosol discharge, ventilation system

## Abstract

Mass COVID-19 infection cases in indoor spaces have been continuously reported since its global outbreak, generating increasing public interest in reducing the spread of the virus. This study considered a situation in which an infected individual continuously releases the virus into the air in a classroom, simulated by continuous injection of NaCl particles ≤ 5 μm, with heater operation during winter. The effects of applying natural ventilation and operating one or two air purifiers on the removal of virus-containing aerosols were experimentally compared and analyzed based on the spatiotemporal changes in NaCl concentration within the classroom. When a heater was operated with all windows shut, operating one and two air purifiers reduced the amount of the aerosol in indoor air by approximately 50 and 60%, respectively, compared to the case with no air purifier. Additionally, when the heater was operated with one or two air purifiers under natural ventilation, the amount of virus-containing aerosol in the air was reduced by 86–88% compared to the case with neither natural ventilation nor air purifier. Because natural ventilation significantly varies with weather conditions and particulate matter concentrations, combining natural ventilation with air purifiers in classrooms during winter needs to be adjusted appropriately.

## 1. Introduction

Since the outbreak of COVID-19, mass viral infection cases in indoor spaces with air conditioner/heater operation have been continuously reported. Viruses that cause respiratory infections, such as the COVID-19 virus, can be transmitted through airborne particles [1]. These virus-containing particles are defined as “droplets” if they are larger than 5 µm and as “droplet nuclei” or “aerosols” if they are smaller than 5 µm. The settling velocity of droplets is generally 30–60 cm/s, and droplet infection may occur through contact or breathing at a distance of 1–2 m. Because the settling velocity of droplet nuclei or aerosols is only 0.06–1.5 cm/s and they are transported along the airflow direction for an extended period, airborne infection is transmitted over a wider range than droplet infection and may infect unspecified individuals [2]. When cooling or heating is performed in indoor spaces during summer or winter, virus particles can be spread over a wide area in the form of aerosols by the airflow from air conditioners or heaters [3]. Therefore, effective control measures are required to remove the viruses generated in indoor spaces and to reduce their spread.

According to the American Society of Heating, Refrigerating and Air-Conditioning Engineers (ASHRAE), increasing the flow rate of the fresh air supplied from the outdoors is important for reducing the probability of airborne infection [4]. Accordingly, ventilation is required to remove indoor infectious agents and provide a comfortable indoor environment. The primary ventilation methods include mechanical and natural ventilation. Humans inhale more toxic substances indoors than outdoors [5], and various toxic substances suspended indoors can adversely affect the human body [6]. Pan et al. [7] stated that ventilation is important because indoor air quality can significantly affect people’s productivity and health. In many countries, e.g., Republic of Korea, U.S., Japan, etc., new buildings are equipped with mechanical ventilation systems due to regulations [8,9]. However, many old buildings built before the regulations came into effect lack such systems. Moreover, according to the U.S. Energy Information Administration, mechanical ventilation systems in buildings consume large amounts of energy, accounting for one-third of the total energy consumption in buildings in developed countries [10]. Therefore, ventilation of indoor spaces through natural ventilation with opened windows could be more effective than mechanical ventilation with high energy consumption [11]. Accordingly, many studies have been conducted on improving indoor air quality through natural ventilation. Allocca et al. [12] studied the application of natural ventilation to reduce building energy consumption and improve indoor air quality using a computational fluid dynamics (CFD) model, and reported that using open windows at different positions to facilitate the inflow of external air from two directions is more effective than introducing external airflow in one direction for the same window opening area. Papakonstantinou et al. [13] analyzed the indoor airflow through single-sided natural ventilation by setting a virtual empty space, and found that the indoor airflow velocity was higher when the windows were opened by 1/2 than by 1/8. Von Grabe et al. [14] evaluated the ventilation efficiency by using various types of windows in an empty space, and determined that horizontal pivot windows are most effective for natural ventilation. Νikolopoulos et al. [15] determined the amount of ventilation required through natural ventilation experiments and CFD analysis for an empty space with double-sided ventilation, and reported that the ventilation efficiency significantly varies with the incident angle of the external airflow. As described, these studies on natural ventilation methods have been conducted mostly for typical buildings or empty spaces rather than specific spaces. 

According to Yonker et al. [16], young people under 22 y can be one of the main sources of viral transmission because they show minimal or no symptoms even when infected. Therefore, various studies have been recently conducted on improving the indoor air quality in school classrooms where many young students may be exposed to the virus. Villanueva et al. [17] assessed the ventilation conditions by measuring the concentrations of CO_2_ and particulate matter (PM; PM_2.5_, PM_10_) in classrooms, and found that the ventilation with opening windows and doors was helpful to adequate ventilation conditions for minimizing the risk of COVID-19 airborne transmission. Stabile et al. [18] evaluated indoor air quality in classrooms by measuring the CO_2_ and particulate matter (PM) concentrations, and suggested applying natural ventilation by opening windows over an extended period to improve the air quality in classrooms, but only during appropriately selected periods to avoid introducing PM into the rooms. Duarte et al. [19] experimentally investigated the improvement of the indoor air quality through natural ventilation in classrooms. They reported that natural ventilation with opened windows is most effective in saving energy and improving the indoor air quality, but the outdoor air temperature must be considered for the thermal comfort of students. Hou et al. [20] studied the ventilation rate through natural ventilation by measuring the CO_2_ concentration in classrooms, and reported that the concentration was effectively reduced by the airflow introduced from the outside during natural ventilation. They also suggested using mechanical ventilation and air purifiers when natural ventilation is not possible. Morawska et al. [21] recommended avoiding crowded spaces, such as classrooms, while reporting on ventilation guidelines to prevent the spread of infectious diseases. They reported that applying natural ventilation with opened windows or installing high-performance air purifiers can help reduce the infection rate. Ji et al. [22] investigated the reduction in indoor PM_2.5_ concentration using air purifiers in an empty space. They revealed that air purifiers can decrease the PM_2.5_ concentration, but failure to replace air purifier filters over an extended period may deteriorate the indoor air quality as the dust accumulated in filters can become a source of toxic substances. Borgese et al. [23] established an indoor air sampling strategy for the detection of COVID-19 virus, and they performed a case study in two kindergartens with opening the windows during outdoor activities of kindergarteners. As demonstrated by many studies conducted during the COVID-19 pandemic, natural ventilation with windows opened and ventilation using air purifiers can effectively reduce the indoor concentrations of toxic substances, and it is the simplest among various ventilation methods. Rivas et al. [24] applied natural ventilation to reduce the infection rate in a space with virus-infected individuals by simulating several virtual scenarios and reported that natural ventilation reduces the virus concentration, but the average concentration can vary from local concentrations based on the internal characteristics of the space. Gil-Baez et al. [25] measured the CO_2_, PM, and organic compound concentrations to evaluate the air quality in classrooms and concluded that natural ventilation practiced during the COVID-19 pandemic can also assure indoor air quality. Park et al. [26] evaluated the effect of natural ventilation on reducing the concentration of the viruses suspended in a school classroom using CO_2_ as a trace gas, and demonstrated that double-sided ventilation was more effective than single-sided ventilation.

In general, as many students spend much time in classrooms, which are enclosed spaces, the number of infected individuals may be quite high without appropriate control measures. However, most previous studies focus on the ventilation rate for the entire classroom, and few studies have explored whether the virus-containing aerosols from infected individuals in a room are effectively removed or discharged in the room based on the ventilation method applied. Therefore, to address the aforementioned issues, this study investigated the injection of an aerosol with particle sizes (≤5 μm) that can cause airborne infection from a specific position in a classroom to simulate the release of virus-containing particles by an infected individual during coughing. Accordingly, the removal efficiency or discharge of the aerosol was experimentally examined according to different ventilation methods applied. A classroom with no mechanical ventilation system was selected for the experiment, and natural ventilation with windows opened as well as ventilation using air purifiers was considered.

## 2. Materials and Methods

**Figure 1:**
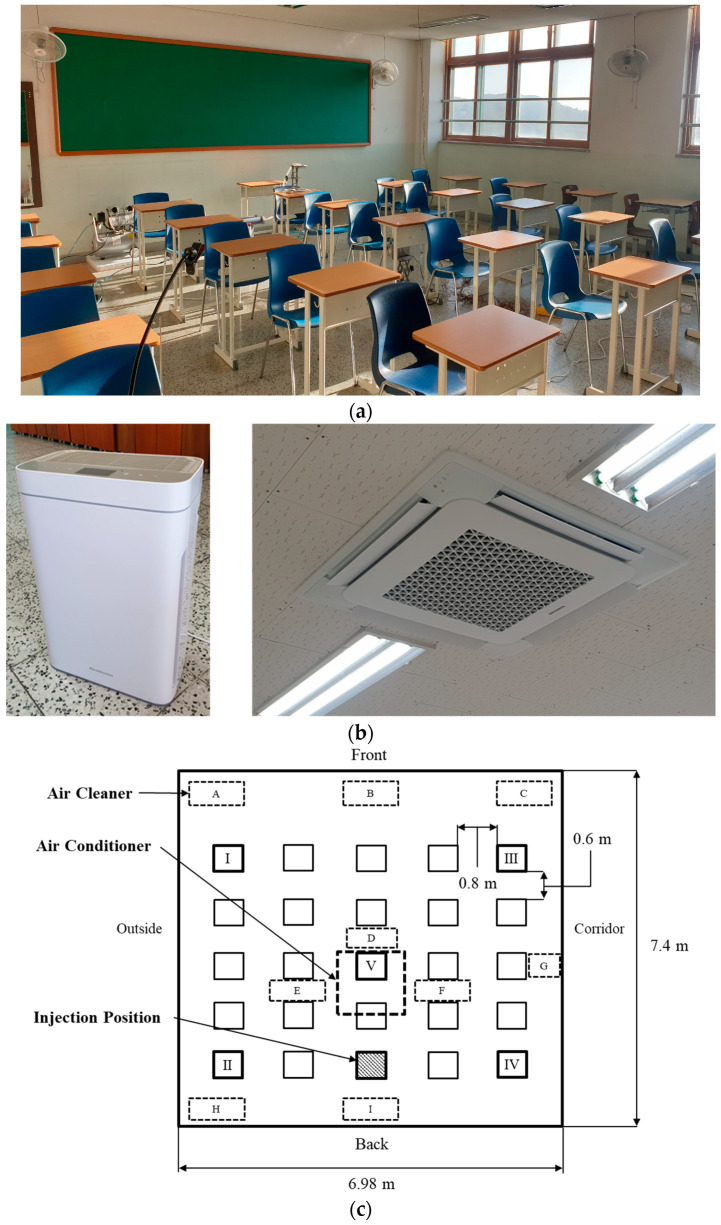
Experimental space selected for the study: (**a**) photograph of the classroom showing the desks and chairs were arranged in a 5 × 5 matrix; (**b**) photographs of the air purifier and the ceiling-type air conditioner installed in the room; (**c**) floor plan of the classroom.

Figure 1 presents the photographs and floor plan of a high school classroom, with dimensions of 7.4 m × 6.98 m × 2.7 m, selected for the experiment. The front side of the classroom faced the north. No mechanical ventilation system was installed in the classroom, and an air purifier was used instead. The air purifier extracts air from both sides and discharges it vertically upwards. Based on the data from the Korea Air Cleaning Association [27], the clean air delivery rate (CADR) required for the area of the target classroom (51.652 m^2^) was determined as 400 m^3^/h; therefore, the air purifier was installed based on this CADR value. However, the practical infection source reduction performance may not reach 400 m^3^/h [28], and the CADR may differ depending on various environmental factors, such as the area and position where the device is installed [27]. Therefore, in this study, the experiment was conducted for two cases where one and two air purifiers were used, respectively. The experiment was performed during winter in December, and the outdoor air temperature ranged from −3 to 1 °C. A four-way cassette fan coil unit (FCU) was installed for heating on the central ceiling of the classroom. Its air discharge angle from the ceiling surface was fixed at 30°, and its air volume was 1080 m^3^/h. The indoor temperature of the four-way FCU control panel was set to 21 °C. In this case, the temperature of the air discharged from the FCU was measured to be 45 °C. To simulate a classroom environment, 25 desks and chairs were arranged in a 5 × 5 matrix in the classroom, and the lateral and longitudinal distances between the desks were 0.8 m and 0.6 m, respectively.

**Figure 2:**
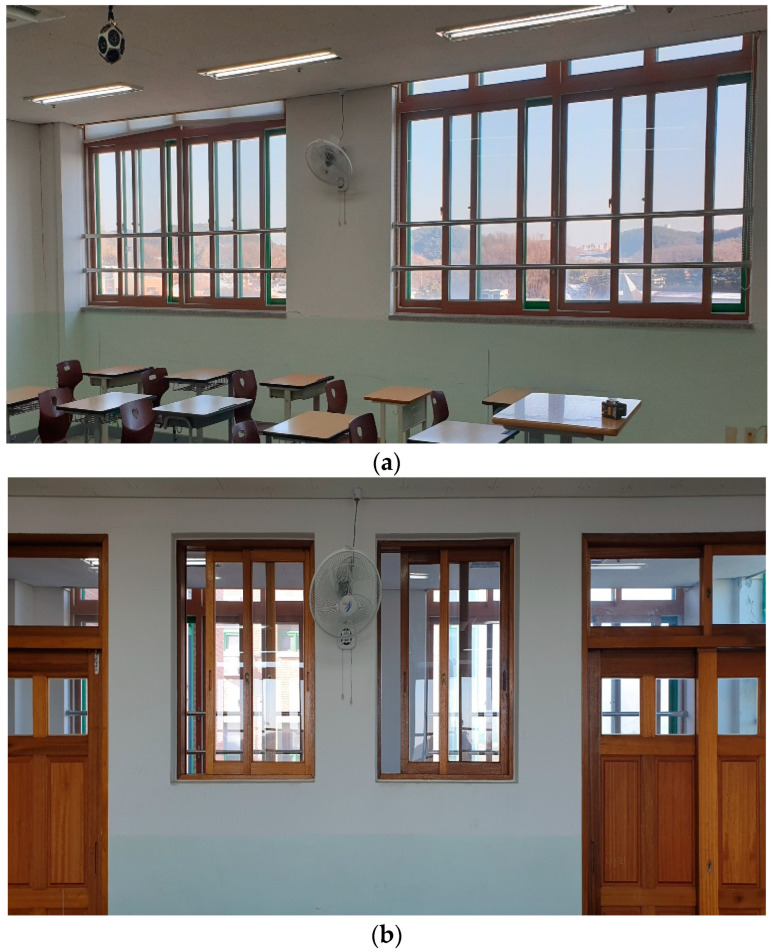
Photographs of the windows in the classroom: (**a**) outside windows; (**b**) corridor windows (and showing the closed front and rear entrances to the classroom).

Figure 2 shows the outside and corridor windows of the classroom. In this study, the use of both air purifiers and natural ventilation with windows opened were considered to effectively remove and discharge the virus-containing aerosol released from an infected individual into the classroom. To reduce the energy loss during the operation of the four-way FCU for heating, all the sliding windows were opened to one-third of their widths, and the front and rear entrances were closed as shown in Figure 2.

**Figure 3:**
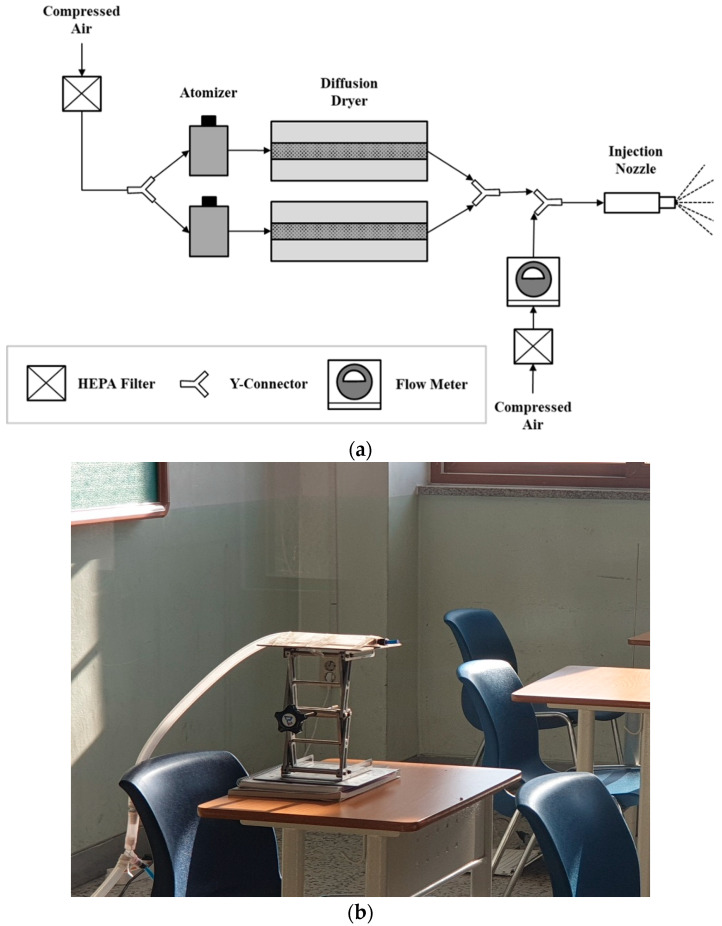
Experimental setup: (**a**) schematic of the particle injection system; (**b**) photograph of the injection nozzle.

Figure 3 shows a schematic diagram and photograph of the experimental setup where an aerosol was continuously injected into the classroom. Atomizers were used to generate an aerosol with a particle size of 5 μm or less, which may cause airborne infection. Compressed clean air was injected into two atomizers containing NaCl solutions dissolved in de-ionized water, and the sprayed droplets passed through two diffusion dryers containing silica gel to remove moisture and generate the NaCl aerosol. The aerosol was then mixed with compressed clean air and injected into the room through an injection nozzle. Considering the saliva droplet discharge rate when a student sneezes, the NaCl aerosol was injected at 15 m/s. Additionally, assuming that an infected individual seated in the middle of the back row of the classroom continues to cough or sneeze, the injection nozzle was installed at the desk corresponding to the injection position indicated in Figure 1c. The installation height of the injection nozzle was set to 1.1 m from the floor considering the position of the mouth of the seated student. To identify the overall diffusion range of the injected NaCl aerosol in the classroom, the particle number concentration was measured at five positions (I to V; Figure 1c) by using five optical particle counters (OPC; Model 1.109, GRIMM Aerosol Technik, Ainring, Bayern, Germany). The OPC measures the size and the number of airborne particles by detecting the intensity of the light scattered by the particles. The height of the sampling probe at each measurement position was set to 1.1 m from the floor, based on the height of the respiratory system of seated high school students. 

In general, a tracer gas was continuously injected into a room and its concentration is measured in a saturated state [29], which is the method primarily used to determine indoor ventilation efficiency. Similarly, for each ventilation method considered in this study, the particle number concentrations at the five positions were measured while the NaCl aerosol was continuously injected until the concentrations at each of the positions converged to a certain value. Additionally, the particle number concentration in the air outside the classroom was measured using another OPC.

In this study, both air purification and natural ventilation were applied to effectively remove and discharge the virus-containing aerosol generated in the classroom. Therefore, 19 cases were investigated according to the number of operated air purifiers and the application of natural ventilation, as shown in Table 1. For heating during winter, the four-way FCU was operated in all the cases. In Cases 1 to 10, no air purifier, or one or two air purifiers were operated while all the windows of the classroom were shut. In Cases 11 to 19, one or two air purifiers were operated while all the outside and corridor windows of the classroom were opened to 1/3 of their widths. When only one air purifier was used, the air purifier was operated at the position of B, C, D, or G by assuming that these positions could represent the center near-wall in the widthwise direction, the corner of the classroom, the central area of the classroom, or the center near-wall in the longitudinal direction, respectively. When two air purifiers were used, the combination of positions for operating two air purifiers were A–C, A–H, B–I, C–H, or E–F, representing two classroom corners in the widthwise direction, two classroom corners in the longitudinal direction, two center near-walls in the longitudinal direction, two classroom corners in the diagonal direction, or two positions in the central area of the classroom, respectively. As Park et al. [26] reported that the ventilation rate by using double-sided ventilation is higher, only the double-sided ventilation condition (that opens windows on both sides of the classroom) was considered in this study.

Because accurately measuring the amount of natural ventilation introduced from the outside through windows can be difficult [26], the amount of natural ventilation was calculated using the following equation, which is commonly used to calculate the amount of natural ventilation when particles are continuously injected into an experimental space under natural ventilation [25,30].

(1) $$C_{p}\left(t\right)=C_{ex}+\frac{E}{Q}+\left(C_{p}\left(0\right)-C_{ex}-\frac{E}{Q}\right)e^{-\frac{Q}{V}t}$$

where *C_p_*(*t*) is the particle number concentration over time (particles/m^3^), *C_p_*(0) is the initial particle number concentration (particles/m^3^), *C_ex_* is the particle number concentration introduced from the outside (particles/m^3^), *E* is the number of the particles injected (particles/h), *Q* is the amount of ventilation (m^3^/h), *V* is the volume of the experimental space (m^3^), and *t* is the time (h). 

**Figure 4:**
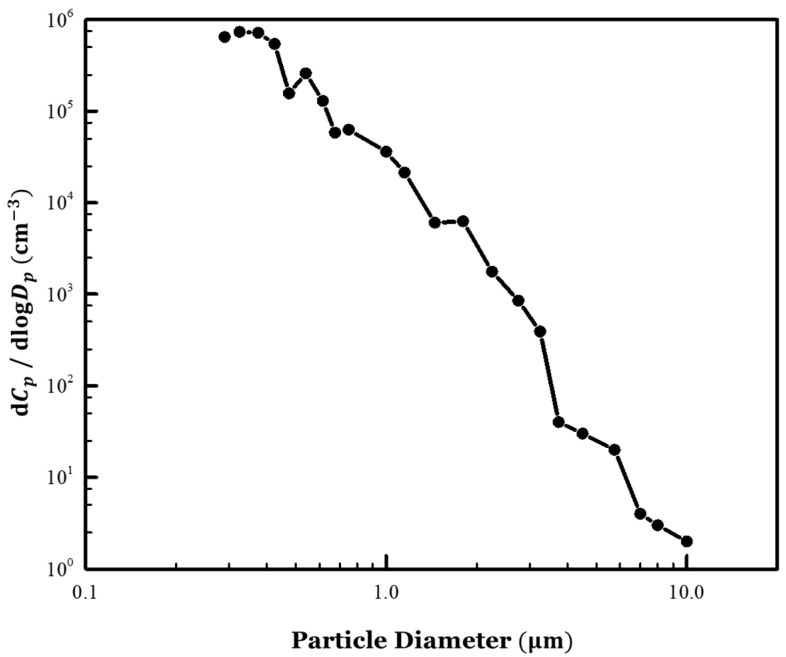
Particle size distribution of the NaCl aerosol injected into the classroom.

Figure 4 shows the particle size distribution measurement results for the NaCl aerosol injected in the classroom. The particle size distribution of the aerosol that can cause airborne infection was found to be well-simulated because most of the generated NaCl particles were <5 µm. In addition, the particle number concentration of the NaCl aerosol injected into the classroom was approximately 90 times higher than that of the outdoor air. Therefore, the influence of the outdoor aerosol introduced into the classroom from the outside by natural ventilation was considered negligible.

## 3. Results and Discussion

**Figure 5:**
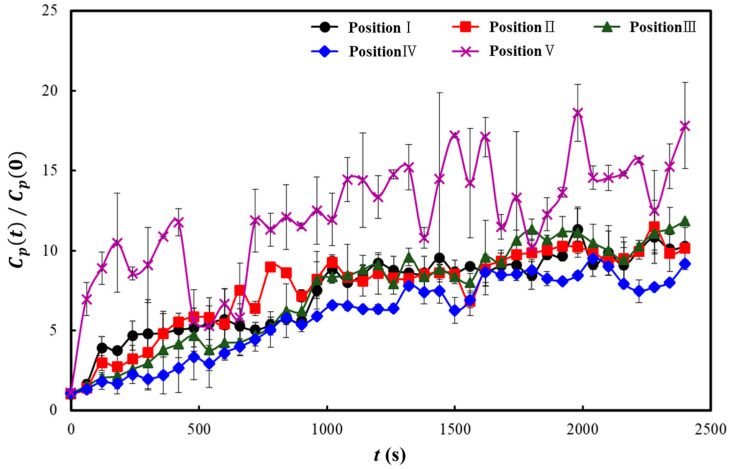
Dimensionless particle number concentrations at measurement positions I to V (the positions are shown in Figure 1c) in the closed condition when only the heater was operated (Case 1), where *C_p_*(*t*)/*C_p_*(0) represents the dimensionless particle number concentration obtained by dividing the particle number concentration over time (*C_p_*(*t*)) by the initial particle number concentration (*C_p_*(0)).

**Figure 6:**
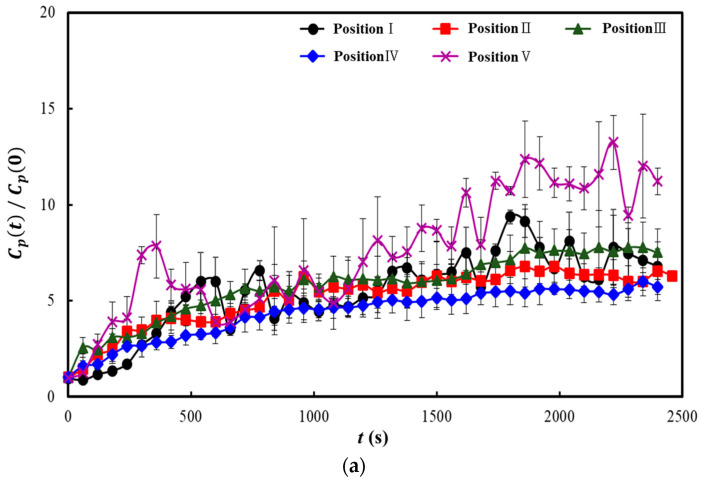

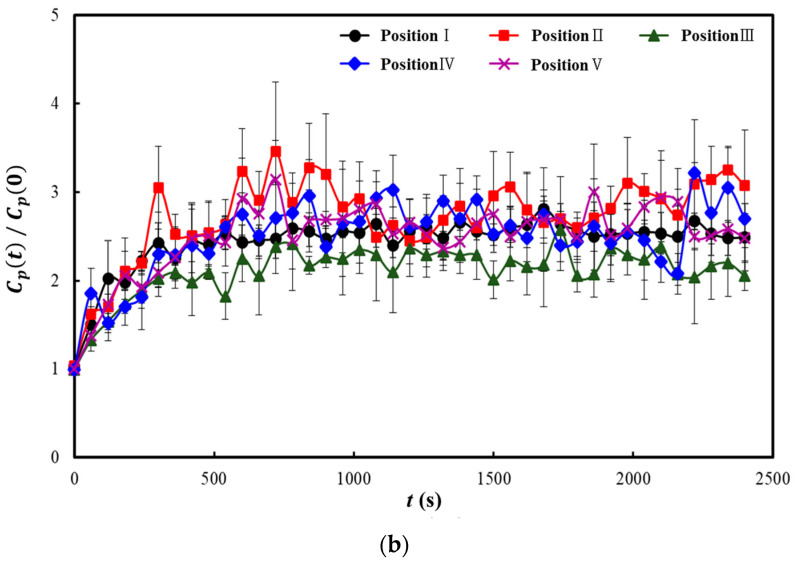
Comparison of the dimensionless particle number concentrations for different measurement positions according to the opening of the windows when one air purifier was operated: (**a**) Case 2; (**b**) Case 11.

**Figure 7:**
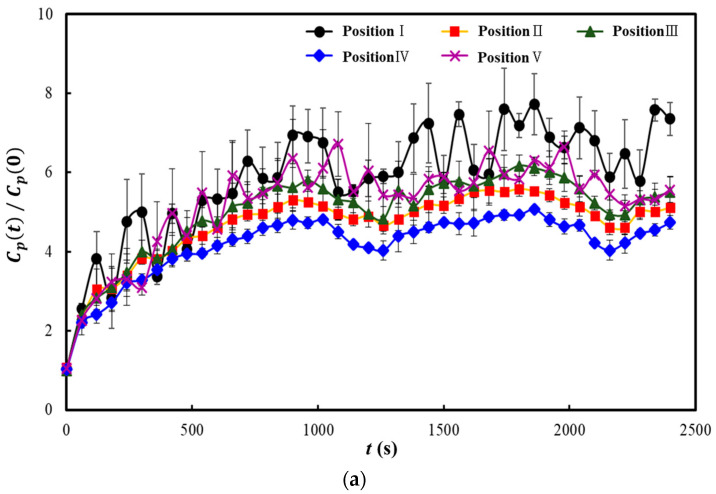

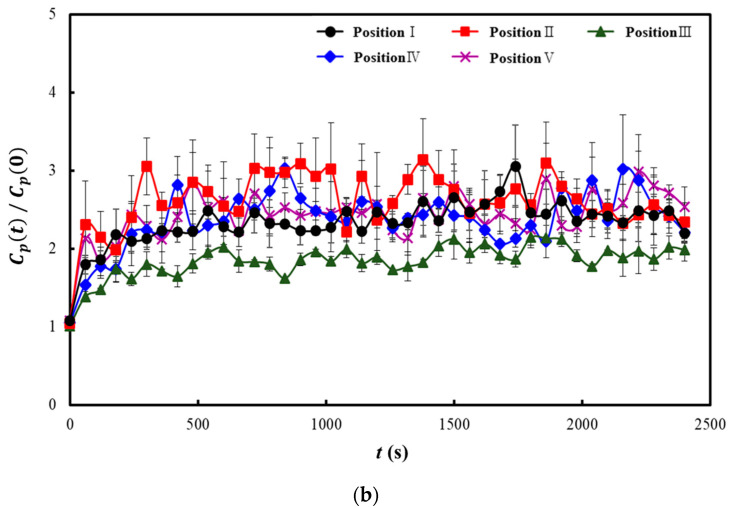
Comparison of the dimensionless particle number concentrations according to the opening of the windows when two air purifiers were operated: (**a**) case 6; (**b**) case 15.

Figure 5, Figure 6 and Figure 7 show the results of some representative cases among the 19 cases considered in this study. The y-axes in these three figures represent the dimensionless particle number concentration obtained by dividing the particle number concentration over time (*C_p_*(*t*)) by the initial particle number concentration (*C_p_*(0)). As the particle number concentration of the NaCl aerosol injected in the classroom was considerably higher than that of the outdoor air, the particle number concentration measured at each position was considered significantly affected by the diffusion results of the NaCl particles injected from the center of the back row of the classroom. Therefore, if the dimensionless particle number concentration increases at a position, the injected NaCl aerosol (i.e., the aerosol containing the virus discharged from the infected individual) had a high probability of being diffused to that position; however, if it decreases, the diffusion probability can be interpreted as low. The particle number concentration was measured for 2400 s because the particle number concentration at each position converged to a certain value within this period for all the 19 cases considered.

Figure 5 shows the results of the experiment when only the four-way FCU for heating was operated, no air purifier was used, and all the windows were shut (Case 1). The dimensionless particle number concentration was up to 18 times higher than the initial particle number concentration at Position V, which was immediately before the NaCl particle injection position, and up to 8–10 times higher at Positions I to IV, which were the four corners of the classroom. This indicates that the virus-containing aerosol discharged through coughing of the infected individual at the rear center of the classroom can be diffused by the airflow of the four-way FCU and remain suspended in air at high particle number concentrations throughout the classroom. 

Figure 6 presents the results of the experiment in which natural ventilation was applied when the heater (four-way FCU) and one air purifier were operated simultaneously. The air purifier was operated at the front-center of the classroom. Figure 6a shows the results when the heater and one air purifier were operated with all windows shut (Case 2). The dimensionless particle number concentration converged to approximately 10 at Position V, and it converged to relatively high values of 5–7 at Positions I to IV. Although these values are lower than those presented in Figure 5, they indicate that the virus-containing aerosol in the classroom cannot be effectively removed by solely operating one air purifier with all windows shut. Figure 6b shows the results when the heater and one air purifier were operated under the natural ventilation condition in which each window on both sides were opened to one-third of its width (Case 11). The dimensionless particle number concentration converged to approximately 2–3 at all positions from I to V. A comparison between the results presented Figure 6b with those in Figure 5 and Figure 6a revealed that the application of natural ventilation along with the operation of one air purifier was more effective in removing the virus-containing aerosol from the classroom, as indicated by the significant decrease in the dimensionless particle number concentration. The amount of ventilation was approximately 3 ACH (air changes per hour) when only one air purifier was operated with all windows shut and approximately 7 ACH when one air purifier was operated with natural ventilation. The amount of ventilation required for a school classroom is 6 ACH; therefore, a sufficient amount of ventilation can be maintained through air purifier operation and natural ventilation but operating only one air purifier without natural ventilation was found insufficient.

Figure 7 shows the results of the experiment in which natural ventilation was applied when the heater and two air purifiers were operated simultaneously on each side at the front of the classroom. Figure 7a shows the results when the heater and two air purifiers were operated with all windows shut (Case 6). In this case, the dimensionless particle number concentration in the classroom converged to approximately 4–7.5; moreover, unlike the previous cases, the concentration was highest at Position 1. According to previous studies related to indoor airflow control [31,32], when several airflows are generated within a space, they are changed into turbulent flows due to the interference between them, and thus, pollutant particles may remain suspended or be re-suspended in a specific area. A comparison between the results of Figure 6a and Figure 7a revealed that operating more air purifiers in the closed condition (i.e., with all windows shut) achieved better removal of the virus-containing aerosol. However, this implies that the aerosol can be diffused to an unintended position or the removal efficiency at a specific position can be insignificant if indoor airflow interference is not considered. Figure 7b shows the results when the heater and two air purifiers were operated under natural ventilation in which all windows on both sides were opened to 1/3 of their widths (Case 15). Herein, the dimensionless particle number concentration converged to approximately 2–2.5; this value is slightly lower compared to that presented in Figure 6b, confirming that operating two air purifiers with natural ventilation can remove the virus-containing aerosol generated indoors more efficiently. When the two air purifiers were operated with all windows shut, the amount of ventilation was found to be approximately 4 ACH, which could not reach the amount of ventilation required for the classroom; however, when the air purifiers were operated with natural ventilation, the required amount of ventilation could be sufficiently ensured because the amount of ventilation was found to be approximately 8 ACH. 

The cases of operating no air purifier (Case 1), one air purifier (Case 2), and two air purifiers (Case 6) were compared under heater operation with all windows shut. Notably, Case 1 showed the highest dimensionless particle number concentration among all 19 cases considered in this study. The dimensionless particle number concentration in Case 2 was approximately 50%, and in Case 6, approximately 40%, of the concentration level observed in Case 1. This finding confirmed that operating one or two air purifiers with all the windows of the classroom shut is effective to some extent in removing the virus-containing aerosol generated indoors; however, the aerosol may be diffused in the classroom at a relatively high particle number concentration. In addition, no significant difference was observed in the reduction in particle number concentration, irrespective of whether one or two air purifiers were used. This was attributed to the inefficient indoor airflow rate compared to the increased total air volume due to the interference between the airflows generated by the two air purifiers and the four-way FCU. Therefore, the optimal operation positions must be determined to minimize the interference between airflows when multiple air purifiers are used in a classroom. Moreover, when the cases of operating one air purifier (Case 11) and two air purifiers (Case 15) under heater operation with all the windows of the classroom opened to 1/3 of the width were compared, the dimensionless particle number concentrations were found to be approximately at 14% and 12% levels, respectively, compared to that in Case 1. The difference in the concentration reduction rate between the cases was not large, but additionally operating air purifiers with natural ventilation can be more helpful in effectively removing or discharging the virus-containing aerosol generated indoors. 

**Figure 8:**
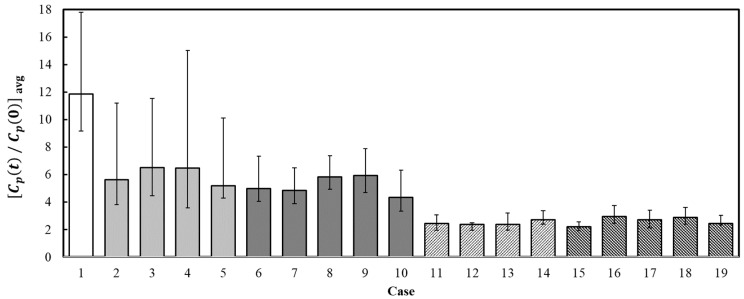
Comparison of the average dimensionless particle number concentrations of each experimental case, with error bars showing the maximum and minimum values among the dimensionless particle concentrations measured at positions of I, II, III, IV and V.

Figure 8 presents a comparison of the average dimensionless particle number concentrations measured at Positions I to V among the 19 cases considered in this study. The dimensionless particle number concentrations for Cases 1, 2, 6, 11, and 15 are shown in Figure 5, Figure 6 and Figure 7, while those for other cases are displayed in Figures S1–S4. Case 1 with only the heater in the closed condition showed the highest value, whereas Case 15 with two air purifiers under natural ventilation and heater operation exhibited the lowest value. Thus, operating one or two air purifiers while the heater was operated with all classroom windows shut (Cases 2 to 10) was able to reduce the particle number concentration of the virus-containing aerosol generated in the classroom by more than half compared to the concentration in Case 1 (with no air purifier). However, the particle number concentration in the classroom can remain high if the virus-containing aerosol is continuously generated (e.g., if the infected individual continues coughing). In addition, a significant difference in the removal efficiencies was observed based on the operating positions of one or two air purifiers. Therefore, the operating positions of air purifiers must be optimized by accounting for the interference between airflows within the classroom. Moreover, operating one or two air purifiers with natural ventilation (Cases 11 to 19) was found to be more effective. In particular, under the application of natural ventilation during heater operation, the average particle number concentration was approximately 57% lower compared to the value measured in the closed condition with no natural ventilation when one air purifier was operated (Cases 2 to 5 vs. Cases 11 to 14) and approximately 52% lower when two air purifiers were operated (Cases 6 to 10 vs. Cases 15 to 19). This indicates that applying natural ventilation with the use of air purifiers is extremely important when a heater is operated in a classroom. Furthermore, operating two air purifiers can further reduce the particle number concentration in the virus-containing aerosol generated in the classroom by approximately 10% on average than operating one air purifier, despite a slight difference depending on the application of natural ventilation. Because the reduction in the particle number concentration of the virus-containing aerosol generated in the classroom varied among Cases 11 to 19, further studies to determine the optimal operating positions is required to maximize the effect of air purifiers even when natural ventilation is applied. In Figure 8, for each case, the error bars show the maximum and minimum values among the dimensionless particle number concentrations measured at the positions of I, II, III, IV, and V. When only one air purifier was operated with all windows closed (Cases 2 to 5), the difference between the average and the maximum dimensionless particle concentration was relatively large, implying that the local air quality distribution under the condition of virus-containing aerosol generation indoors can be extremely non-uniform. However, by operating two air purifiers with all windows closed (Cases 6–10), the error bars became smaller than those for the cases of one purifier operation without natural ventilation, meaning that the problem of non-uniform local air quality distribution can be somewhat alleviated. Moreover, by operating one or two air purifiers with applying natural ventilation (Cases 11–19), the sizes of error bars shortened further, signifying that the local air quality distribution under the condition of virus-containing aerosol generation indoors can be more uniform compared to the cases of air purifier operation without natural ventilation. Based on the results shown in Figure 8, operating air purifiers combined with natural ventilation by opening windows even slightly was inferred to be effective in reducing the particle number concentration of the virus-containing aerosol generated indoors during heater (four-way FCU) operation. As the ventilation rate under natural ventilation may significantly vary based on weather conditions and cases in which windows cannot be opened due to the very low outdoor air temperature or high PM concentrations, operating one or more air purifiers (preferably several units) at all times in a classroom is required. In addition, the optimal air purifier operating positions must be determined according to the classroom environment. 

## 4. Conclusions

This study experimentally analyzed a situation in which an individual infected with COVID-19 continues to release virus particles through coughing or sneezing in a classroom with no mechanical ventilation system, and simulated this environment using NaCl particles ≤5 μm, which is the particle size that can cause airborne infection. The effects of applying natural ventilation and one or two air purifiers at different positions were investigated under heater (four-way FCU) operation during winter, and accordingly, 19 cases were considered. For natural ventilation, each window in the classroom was opened to 1/3 of its width. Among the 19 cases, Case 1 with the heater, all windows shut, and no air purifier operation exhibited the highest concentration. Thus, this was selected as the reference case with which the effects of other ventilation methods were compared and evaluated. Compared to the particle number concentration in Case 1, when the heater was operated with all windows shut, the concentration of the virus-containing aerosol discharged indoors was reduced by approximately 50% using one air purifier and by approximately 60% with two air purifiers. Moreover, when one or two air purifiers were operated under natural ventilation, the particle number concentration decreased by 86% or 88%, respectively, compared with Case 1, confirming that operating air purifiers with natural ventilation is highly effective. Regardless of the application of natural ventilation, the use of two air purifiers than one could further reduce the particle number concentration of the virus-containing aerosol discharged indoors. In addition, the effect of air purifiers was different based on their operating positions, confirming the need to determine the optimal operating positions for the air purifiers. The experimental results confirmed that a remarkable reduction effect can be achieved in a classroom with heater operation in winter by using air purifiers while applying natural ventilation by opening (even slightly) all windows on both sides of the room. As natural ventilation rates vary depending on weather conditions, such as the outdoor air temperature, wind direction, wind speed, and particulate matter (PM) concentrations, operating one or more air purifiers in a classroom with no mechanical ventilation system is considered necessary to maintain the indoor air quality. Operating two air purifiers compared to one was found to be slightly more effective in removing or discharging the virus-containing aerosol discharged in a classroom; however, further research for optimizing the operating positions of these air purifiers is required to maximize the effect. Currently, in Korea, the Korea Disease Control and Prevention Agency (KDCA) recommends that schools apply natural ventilation to classrooms by opening both outside and corridor-side windows as frequently as possible, in order to take preventive measures against COVID-19 spread. The results of this study are significant in the sense that the guideline of the KDCA can be effective in lowering the concentration of virus-containing aerosol generated indoors. Furthermore, the appropriate ventilation rates must be systematically identified by applying natural ventilation and air purifiers under various weather conditions, and accordingly, guidelines for various scenarios must be established to ensure good indoor air quality. 

## Figures and Tables

**Table 1:** Experimental conditions according to the application of natural ventilation and the operation of air purifiers in the classroom.

Case	Air Purifier		Four-Way Cassette Fan Coil Unit	Outside Window	Corridor Window
	Quantity	Position as Shown in Figure 1c			
1	0	-	On	Close	Close
2	1	B	On	Close	Close
3	1	C	On	Close	Close
4	1	G	On	Close	Close
5	1	D	On	Close	Close
6	2	A and C	On	Close	Close
7	2	B and I	On	Close	Close
8	2	C and H	On	Close	Close
9	2	A and H	On	Close	Close
10	2	E and F	On	Close	Close
11	1	B	On	Open	Open
12	1	C	On	Open	Open
13	1	G	On	Open	Open
14	1	D	On	Open	Open
15	2	A and C	On	Open	Open
16	2	B and I	On	Open	Open
17	2	C and H	On	Open	Open
18	2	A and H	On	Open	Open
19	2	E and F	On	Open	Open

## Data Availability

Not applicable.

## Supplementary Materials

The following supporting information can be downloaded at: https://www.mdpi.com/article/10.3390/toxics10100573/s1, Figure S1. Comparison of the dimensionless particle number concentrations for different measurement positions when one air purifier was operated with all windows closed: (a) Case 3; (b) Case 4; (c) Case 5. Figure S2. Comparison of the dimensionless particle number concentrations for different measurement positions when two air purifiers were operated with all windows closed: (a) Case 7; (b) Case 8; (c) Case 9; (d) Case 10. Figure S3. Comparison of the dimensionless particle number concentrations for different measurement positions when one air purifier was operated with all windows open: (a) Case 12; (b) Case 13; (c) Case 14. Figure S4. Comparison of the dimensionless particle number concentrations for different measurement positions when two air purifiers were operated with all windows open: (a) Case 16; (b) Case 17; (c) Case 18; (d) Case 19.
