# Supplementary material for: Natural Ventilation and Air Purification for Effective Removal of Airborne Virus in Classrooms with Heater Operation
Source: Toxics. 2022 Sep 30;10(10):573. doi: 10.3390/toxics10100573 (PMC9607292; doi:10.3390/toxics10100573)
Supplement: Supplementary file 1 [file toxics-10-00573-s001.zip › toxics-1916745-supplementary.pdf]

Supplemental Materials

Natural Ventilation and Air Purification for Effective Removal of Airborne Virus in Classrooms with Heater Operation

Su-Hoon Park[1], Se-Jin Yook[1,*], Hyun Bon Koo[2,*]

[1] School of Mechanical Engineering, Hanyang University, Seoul 04763, Korea
[2] Department of Structural Engineering Research, Korea Institute of Civil Engineering and Building Technology, Goyang-si 10223, Gyeonggi-do, Korea
*Correspondence: ysjnuri@hanyang.ac.kr (S.-J.Y.), mygoomy@kict.re.kr (H.B.K.)

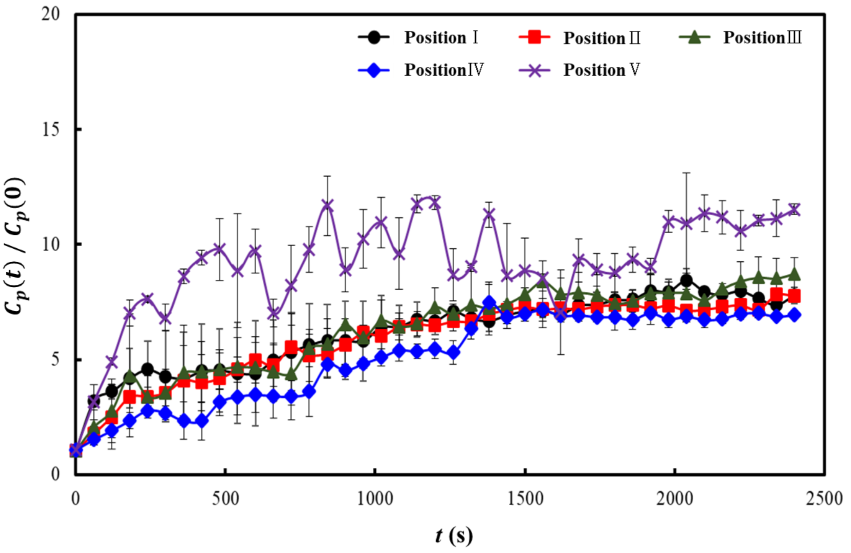

(a)

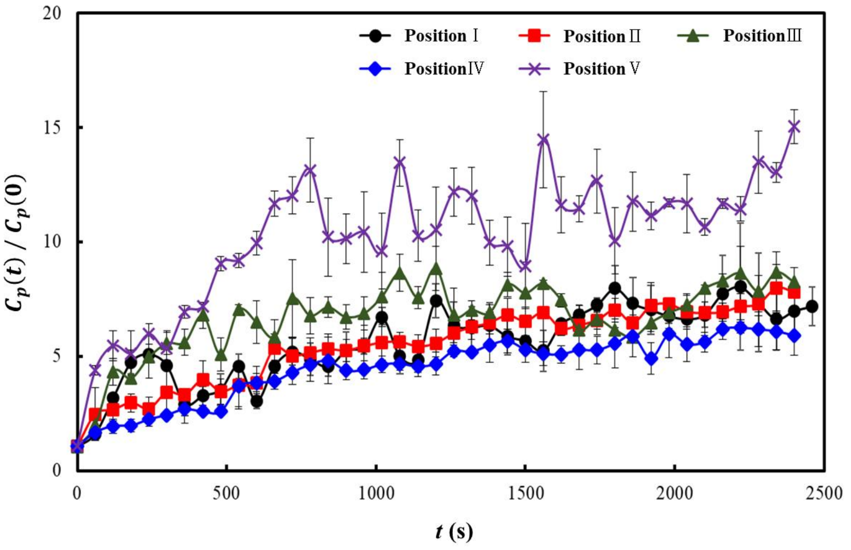

(b)

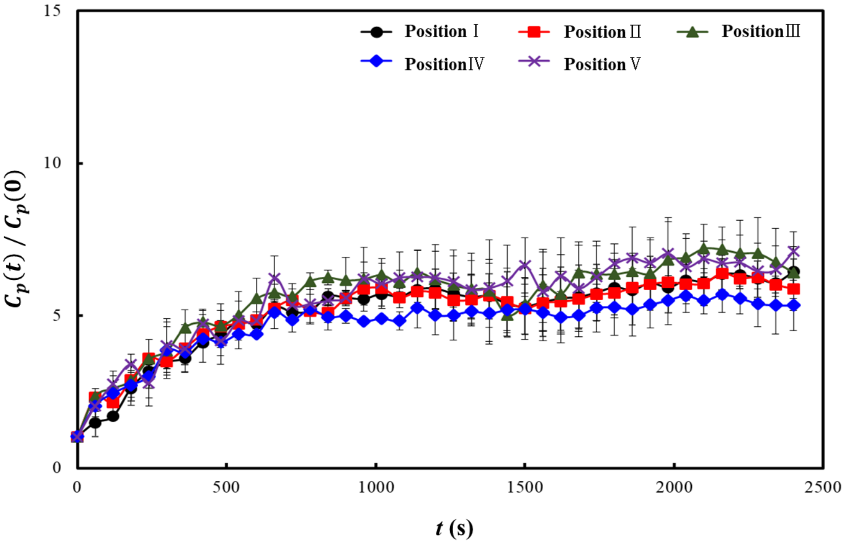

(c)

Figure S1. Comparison of the dimensionless particle number concentrations for different measurement positions when one air purifier was operated with all windows closed: (a) Case 3; (b) Case 4; (c) Case 5.

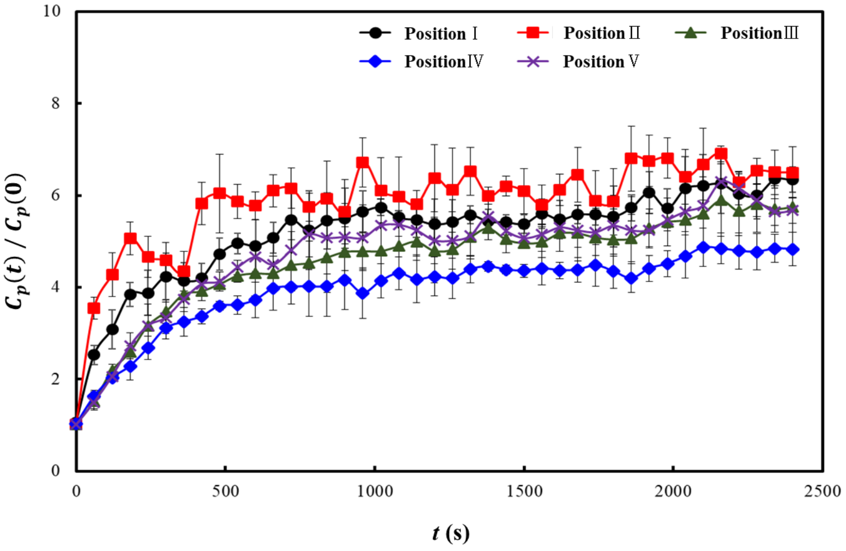

(a)

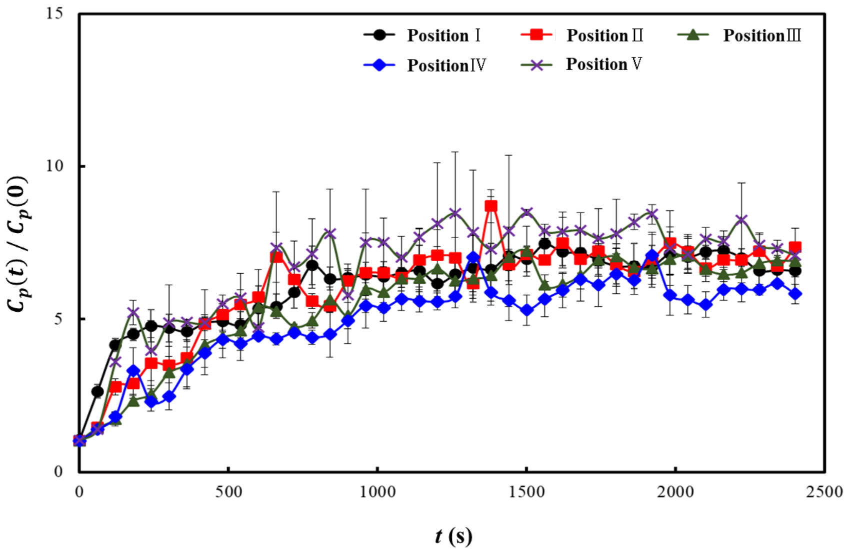
Position Ⅰ
Position Ⅱ
Position Ⅲ
Position Ⅳ
Position Ⅴ
$C_p(t) / C_p(0)$
t (s)
0
5
10
15
0
500
1000
1500
2000
2500

(b)

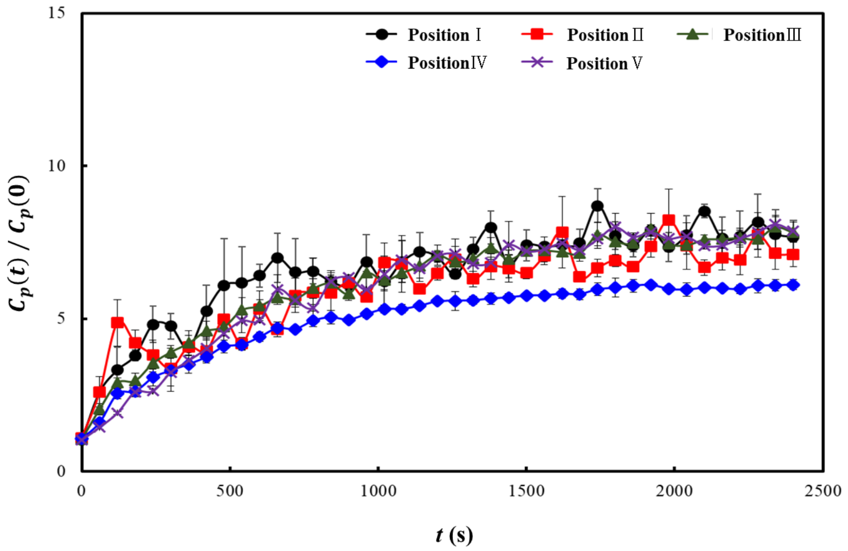
Position Ⅰ
Position Ⅱ
Position Ⅲ
Position Ⅳ
Position Ⅴ
$C_p(t) / C_p(0)$
t (s)
0
5
10
15
0
500
1000
1500
2000
2500

(c)

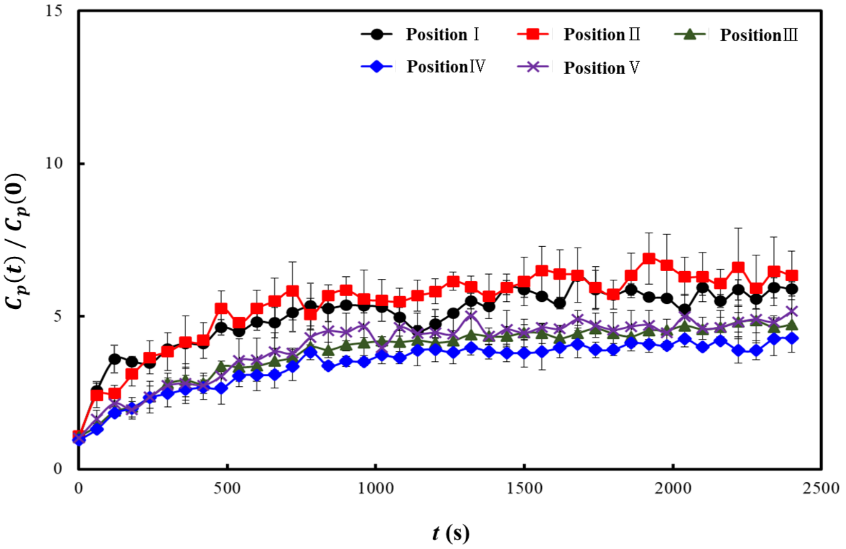

(d)

Figure S2. Comparison of the dimensionless particle number concentrations for different measurement positions when two air purifiers were operated with all windows closed: (a) Case 7; (b) Case 8; (c) Case 9; (d) Case 10.

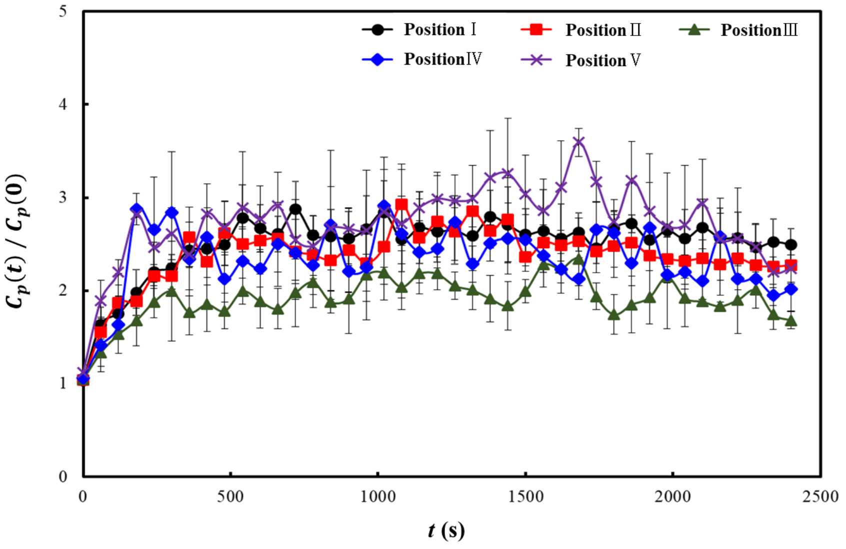

(a)

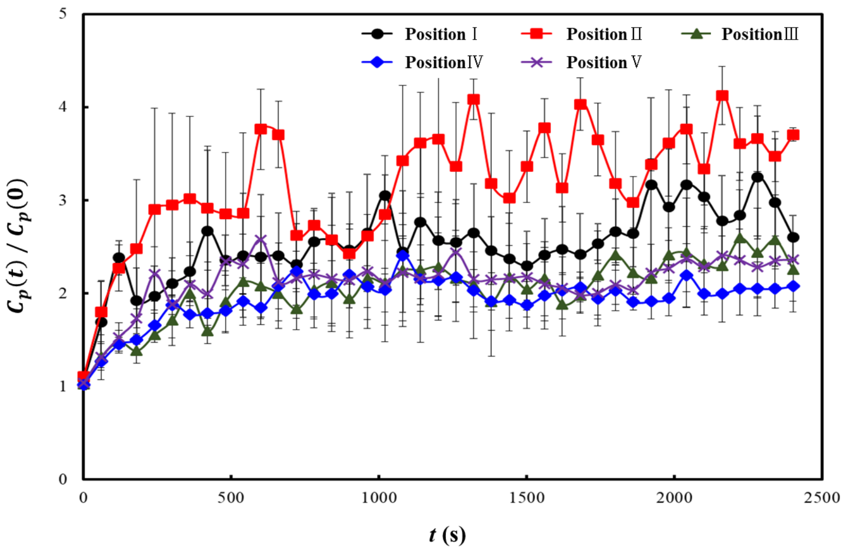

(b)

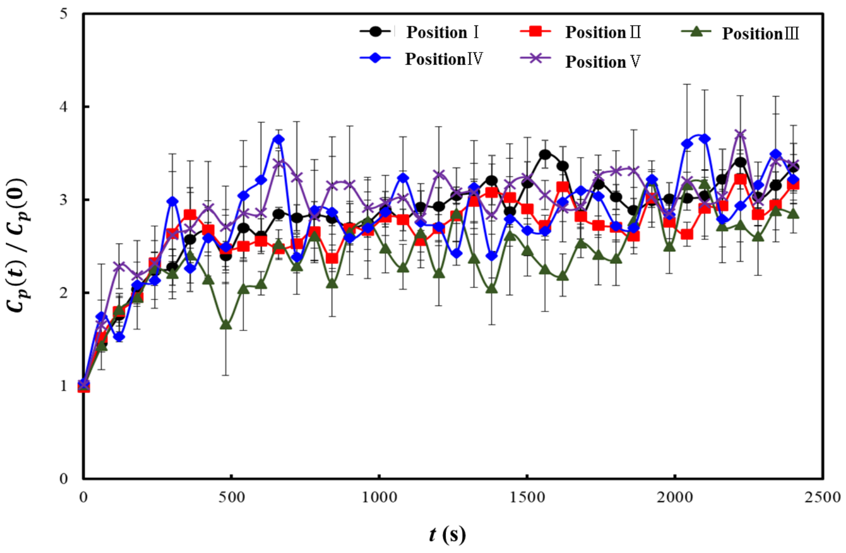

(c)

Figure S3. Comparison of the dimensionless particle number concentrations for different measurement positions when one air purifier was operated with all windows open: (a) Case 12; (b) Case 13; (c) Case 14.

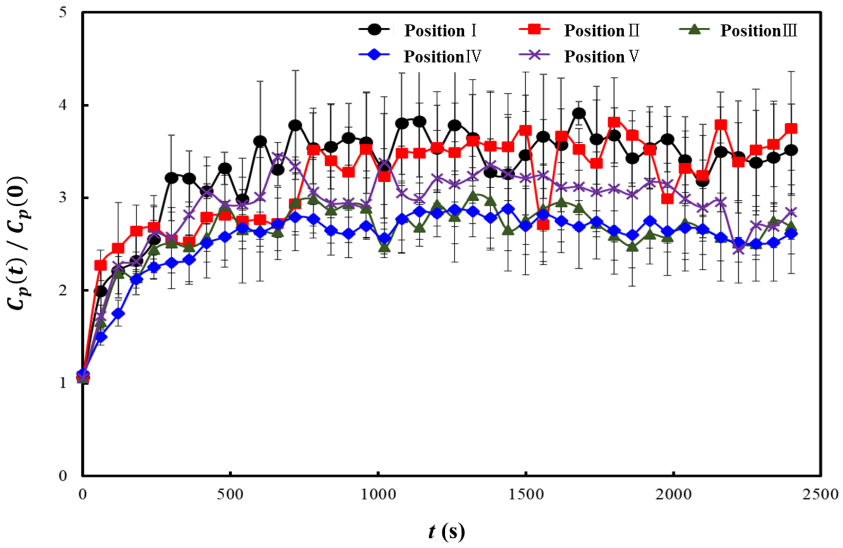

Position Ⅰ
Position Ⅱ
Position Ⅲ
Position Ⅳ
Position Ⅴ
$C_p(t) / C_p(0)$
t (s)

(a)

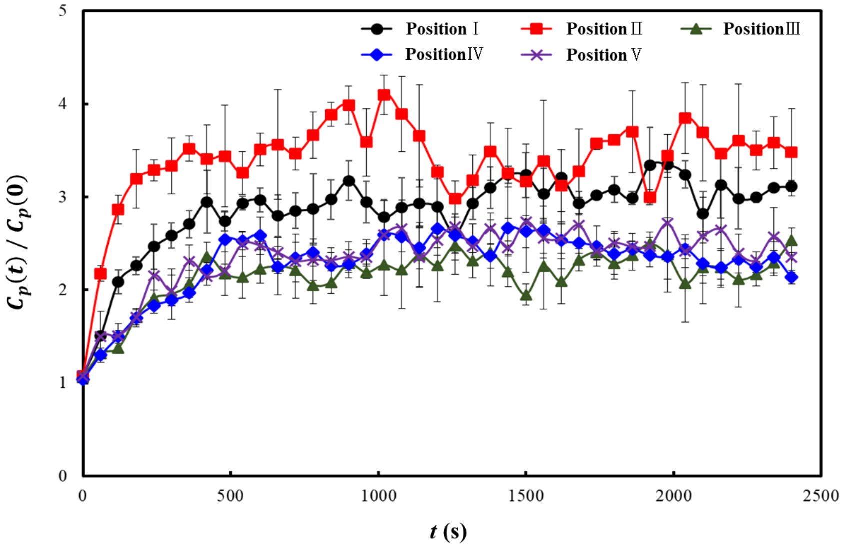

Position Ⅰ
Position Ⅱ
Position Ⅲ
Position Ⅳ
Position Ⅴ
$C_p(t) / C_p(0)$
t (s)

(b)

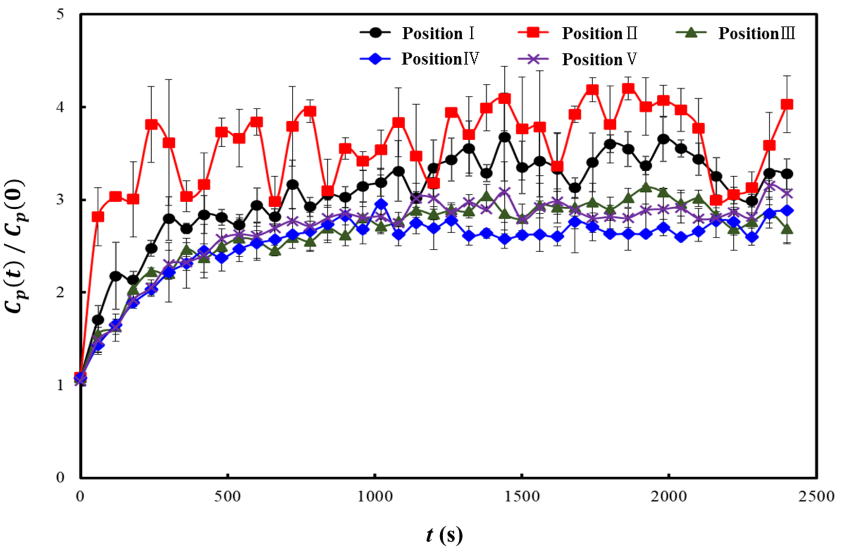

(c)

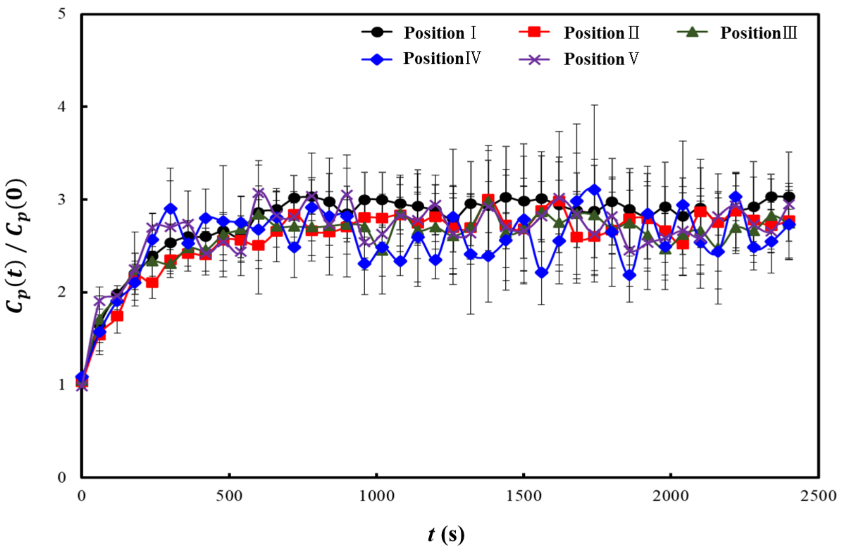

(d)

Figure S4. Comparison of the dimensionless particle number concentrations for different measurement positions when two air purifiers were operated with all windows open: (a) Case 16; (b) Case 17; (c) Case 18; (d) Case 19.
